# Intrauterine Growth Restriction Alters Postnatal Hippocampal Dentate Gyrus Neuron and Microglia Morphology and Cytokine/Chemokine Milieu in Mice

**DOI:** 10.3390/life14121627

**Published:** 2024-12-09

**Authors:** Frank A. Strnad, Ashley S. Brown, Matthew Wieben, Emilio Cortes-Sanchez, Megan E. Williams, Camille M. Fung

**Affiliations:** 1 Division of Neonatology, Department of Pediatrics, University of Utah School of Medicine, Salt Lake City, UT 84108, USA; frank.strnad@hsc.utah.edu (F.A.S.); ashleys.brown@hsc.utah.edu (A.S.B.); matthew.wieben@hsc.utah.edu (M.W.); 2 Huntsman Cancer Institute, Salt Lake City, UT 84112, USA; emilio.cortes-sanchez@hci.utah.edu; 3 Department of Neurobiology, University of Utah School of Medicine, Salt Lake City, UT 84112, USA; megan.williams@neuro.utah.edu

**Keywords:** hypertensive diseases of pregnancy, uteroplacental insufficiency, intrauterine growth restriction, fetal growth restriction, hippocampus, dentate gyrus neurons, microglia, learning and memory

## Abstract

Infants born with intrauterine growth restriction (IUGR) have up to a five-fold higher risk of learning and memory impairment than those with normal growth. Using a mouse model of hypertensive diseases of pregnancy (HDP) to replicate uteroplacental insufficiency (UPI), we have previously shown that UPI causes premature embryonic hippocampal dentate gyrus (DG) neurogenesis in IUGR offspring. The DG is a brain region that receives the first cortical information for memory formation. In the current study, we examined the postnatal DG neuron morphology one month after delivery (P28) using recombinant adeno-associated viral labeling of neurons. We also examined DG microglia’s morphology using immunofluorescent histochemistry and defined the hippocampal cytokine/chemokine milieu using Luminex xMAP technology. We found that IUGR preserved the principal dendrite lengths but decreased the dendritic branching and volume of DG neurons. IUGR augmented DG microglial number and cell size. Lastly, IUGR altered the hippocampal cytokine/chemokine profile in a sex-specific manner. We conclude that the prematurely-generated neuronal progenitors develop abnormal morphologies postnatally in a cell-autonomous manner. Microglia appear to modulate neuronal morphology by interacting with dendrites amidst a complex cytokine/chemokine environment that could ultimately lead to adult learning and memory deficits in our mouse model.

## 1. Introduction

IUGR, defined as a fetus’ inability to gain weight based on his/her genetic potential, complicates up to 10% of human pregnancies [1]. After prematurity, IUGR is the leading cause of perinatal morbidity and mortality in neonatal medicine [2]. Despite advances in neonatal care, infants with IUGR remain at high risk of neurodevelopmental delays that include learning and memory impairment [3,4]. The most common cause of IUGR in developed countries is hypertensive diseases of pregnancy (HDP), which includes gestational hypertension, preeclampsia, eclampsia, and chronic hypertension with superimposed preeclampsia [5,6]. Other maternal conditions such as cigarette smoking, chronic kidney disease, and diabetes mellitus could also precipitate HDP. Maternal hypertension vasoconstricts a normally low-resistance placenta, causing uteroplacental insufficiency (UPI) and decreasing oxygen and nutrient transfer to the fetus. The fetus adapts to chronic hypoxia by shunting blood flow to vital organs such as the brain [7]. Despite such an adaptation, infants born with IUGR carry a five-fold higher risk of learning and memory impairment than those with normal growth [8,9].

The mechanisms behind learning and memory impairment remain poorly defined. This is partly because we lack an understanding of the fetal brain changes in the adverse UPI environment that sets the IUGR offspring up for future deficits. The majority of research in this field has focused on postnatal neuronal phenotype within the hippocampus [10,11,12], a brain region charged with learning and memory formation. What is known is that IUGR causes postnatal hippocampal neuronal loss, which culminates in hippocampal volume loss, and there is a direct correlation between the severity of the volume loss and learning and memory deficits. However, the mechanisms and timing of how UPI perturbs embryonic neurogenesis and neuronal progenitor cell maturation to result in postnatal neuronal and hippocampal volume loss are less explored. 

Our laboratory has chosen to address the fetal origins of adult learning and memory deficits by using our own mouse model of HDP/UPI to generate IUGR offspring that can be examined longitudinally [13]. We have previously shown that, within three days of HDP/UPI, the IUGR hippocampal dentate gyrus (DG) shows premature embryonic neurogenesis, which persists until delivery [14]. Premature neurogenesis occurs at the expense of decreased neural stem cell (NSC) proliferation, causing concurrent NSC depletion. The DG is a critical region because DG dendrites receive incoming cortical information from the entorhinal cortex into the hippocampus for memory formation [15,16,17]. DG axons then send outgoing information to Cornu Ammonis (CA) 3 neurons in a unidirectional manner to consolidate memory. Any perturbation in DG neuron maturation could alter the ability to form new memories. Our IUGR mouse offspring in this model indeed exhibit adult recognition memory deficits demonstrated by a negative discrimination index via novel objection recognition and lack of freezing time both in contextual and cued fear conditioning when compared to appropriately-grown offspring [14]. Interestingly, our IUGR female offspring have the smallest hippocampal volumes in the juvenile ages and worse memory deficits as adults compared to IUGR males [14].

Given the known learning and memory deficits in this model, the current study strives to examine the postnatal sequelae of premature embryonic DG neurogenesis by examining dendrite development one month after birth, an active time of dendritogenesis and synaptogenesis in rodents [18]. Because microglia have been recognized to play important roles in the formation and function of neuronal circuits in recent years [19], we additionally sought to characterize DG microglia morphology and define the hippocampal cytokine and chemokine milieu as part of our microglial characterization. We recognize that multiple cell types within the DG produce cytokines and chemokines and are not microglial-specific, but we have chosen a panel of cytokines and chemokines that are typically associated with microglial activity. We hypothesize that the DG neuronal progenitors that are generated prematurely in embryogenesis will develop an abnormal dendritic morphology in postnatal life that is modulated partly by microglia and their inflammatory environment. We discovered that IUGR hippocampal DG dendrites of both sexes have reduced branching and volume one month after birth in association with increased microglial number, cell size, and ramifications compared to sham hippocampal DG. We additionally discovered that the IUGR hippocampal cytokine/chemokine milieu has a sexually divergent expression that likely influences neuron–microglia interactions.

## 2. Materials and Methods

### 2.1. Mouse Model of HDP/UPI/IUGR

All procedures involving mice were approved by the University of Utah Animal Care and Use Committee. C57BL/6J mice (strain # 00064, The Jackson Laboratory, Bar Harbor, ME, USA) were housed in cages of up to five mice of the same sex, with standard vivarium conditions of 65–75 degrees Fahrenheit, 40–60% humidity, and a 12:12 light:dark cycle. We used our well-established mouse model of HDP that produces UPI to generate symmetrical IUGR mouse offspring [13]. Briefly, we performed timed-mating on C57BL/6J mouse dams (strain # 00064, The Jackson Laboratory, Bar Harbor, ME, USA) with the post-coitus plug seen on the next morning denoted as day 0.5 of pregnancy. On embryonic day (E) 12.5, pregnant dams received intraperitoneal injections of ketamine (80–100 μg/g mouse) and xylazine (7.5–16 μg/g mouse). After adequate sedation, we made a 1 cm incision at the right hip and a micro-osmotic pump (#1007D, Alzet, Durect Corporation, Cupertino, CA, USA) containing either vehicle (0.5% ethanol = sham or appropriately grown group) or a thromboxane A_2_-analog, U-46619 (#16450, Cayman Chemical Company, Ann Arbor, MI, USA, MI = IUGR group) dissolved in the vehicle was inserted into the retroperitoneal space. The incision site was sewn up. Both sham and IUGR mouse litters were delivered naturally and were cross-fostered to dams without prior surgery. We defined pups who have suffered IUGR as ≤10% in birth weight based on the sham cohort, equating to ≤1.266 g [13].

### 2.2. Recombinant Adeno-Associated Virus (rAAV) Production to Label Hippocampal Glutamatergic DG Neurons

**Figure 1:**
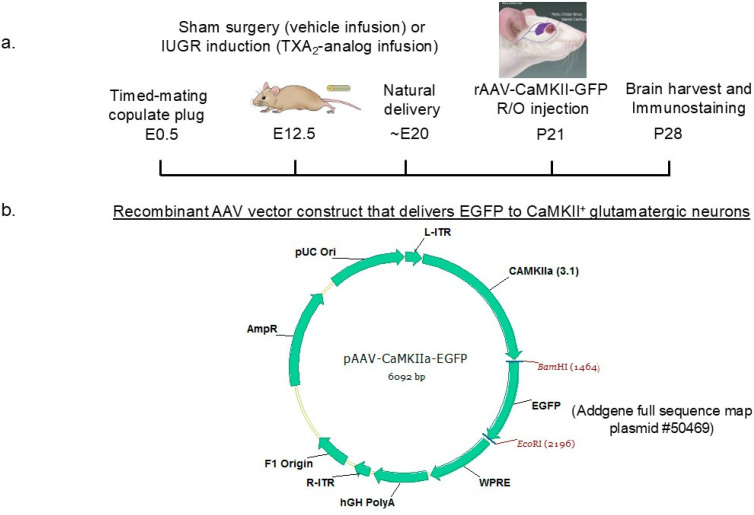
(**a**) Schematic diagram of the experimental timeline and (**b**) a cartoon of the recombinant adeno-associated vector (rAAV) construct that labels CaMKII^+^ glutamatergic neurons with an enhanced green fluorescent protein (EGFP). Mating mouse pairs were placed into the same cage the night before. A copulated plug the next morning denoted embryonic day (E) 0.5 of pregnancy. We performed sham surgery or IUGR induction via micro-osmotic pump implantation of either a vehicle (0.05% ethanol) or a thromboxane A_2_ (TXA_2_)-analog infusion at E12.5. All pups were delivered naturally and cross-fostered to unmanipulated mouse dams. On postnatal day (P) 21, we sedated and injected rAAV-EGFP vectors into the retro-orbital veins. Brains were harvested at P28 for immunohistochemistry.

rAAV vector particles were generated at the University of Utah Drug Discovery Core by transfecting an AAV vector plasmid containing a transgene encoding an enhanced green fluorescent protein (EGFP) targeting Ca2^+^/calmodulin-dependent protein kinase II^+^ (CaMKIIa^+^) glutamatergic neurons, (pAAV-CaMKIIa-EGFP, #50469, Addgene, Watertown, MA, USA), an AAV2 helper plasmid, and an adenovirus helper plasmid into HEK293 cells following biosafety level 2 procedures. The resulting viral titer was measured at 5.02 × 10^12^ viral genome copies/mL. Once sham and IUGR mice were weaned on postnatal (P) day 21, they were anesthetized with halothane in a bell jar until respiratory rate slowed, and a retro-orbital injection containing 30 μL of a mixture of sterile 10x phosphate-buffered saline, fast green dye, AAV virus, and deionized water was made. After one week of viral infection, P28 mouse brains were harvested for immunofluorescent staining (n = 8/group/sex, Figure 1).

### 2.3. P28 Mouse Brain Perfusion and Immunofluorescent Histochemistry for GFP^+^ DG Neurons/Dendrites and Iba1^+^ Microglia

P28 sham and IUGR mice were sedated with ketamine and xylazine. Once adequate anesthesia was achieved, as indicated by a lack of withdrawal by toe pinch, a thoracotomy was performed to expose the heart. A 25 gauge needle was inserted into the left ventricle, the right atrium was snipped, and the mouse was perfused with normal saline until the blood exiting the right atrium was clear, followed by 4% paraformaldehyde (PFA). Brains were extracted and post-fixed with an additional 4% PFA at 4° Celsius (C) overnight. The brains were then cryoprotected with 10% followed by 30% sucrose and sectioned on a vibratome at 50 μm thickness. All coronal sections from the beginning of the dorsal to ventral hippocampus were collected and placed individually into 24-well plates containing sterile PBS. Plates were wrapped with parafilm and stored at 4 °C until staining. 

Coronal sections containing the dorsal hippocampus were identified, and every third section was used to complete one set of staining per brain to achieve systematic random sampling. On average, 15–18 brain sections were included for each dorsal hippocampus. We blocked all sections with 10% normal goat serum (NGS) in PBS-Tween (PBS-T) for 90 min at room temperature. We then incubated all sections with 1:4000 dilution of chicken polyclonal IgY anti-GFP (#ab 13970, Abcam, Waltham, MA, USA) and 1:400 dilution of rabbit polyclonal IgG anti-Iba1 (ionized calcium-binding adaptor molecule 1, #019-19741, Fujifilm Wako, Osaka, Japan) antibodies in 4% NGS/PBS-T overnight at 4 °C. After washing, sections were incubated with 1:1000 dilution of goat anti-chicken IgY Alexa Fluor 488 (#A11039, Thermo Fisher Scientific, Waltham, MA, USA) and 1:400 goat anti-rabbit IgG Alexa Fluor 568 (#A11011, Thermo Fisher Scientific) secondary antibodies in 4% NGS/PBS-T for 2 h at room temperature. We used a 1:5000 dilution of 4′,6-diamidino-2-phenylindole dihydrochloride (DAPI) (#D1306, Thermo Fisher Scientific) at 1 μg/mL in PBS for nuclear staining. After thorough washing, tissues were mounted on glass slides, cover-slipped using Fluoromount-G (#0100-01, Southern Biotech, Birmingham, AL, USA), and left to cure in a light-protected slide box overnight before imaging.

### 2.4. Imaging and Analysis of GFP^+^ Neurons/Dendrites and Iba^+^1 Microglia

All brain sections were imaged using a laser-scanning confocal microscope (Olympus, Tokyo, Japan) with either a 20x, 40x, or 60x objective lens and Fluoview FV1000 software (Olympus). We used 20x magnification to visualize the entire dorsal hippocampus DG and 40x and 60x magnifications for greater delineation of cell morphologies for quantification. Each hippocampal section was imaged at 0.5 μm z-stacks to allow for dendrite tracing. Once images were obtained, we used NIH ImageJ software to split the multi-channel images into blue (DAPI), green (GFP), and red channels (568) individually. To measure the principal dendrite lengths of GFP^+^ DG neurons, we used the ImageJ FIJI Simple Neurite Tracer plugin to trace the longest dendrite from cell soma to the border of the entorhinal cortex. With our rAAV labeling protocol, we aimed to label no more than 20 DG neurons per section after optimization. It was important not to label all neurons as this would make dendrite tracing difficult. All traces were saved, and raw path lengths were collected. To measure the volume of the dendritic projections, we used the saved stacked images and ran the following ImageJ macro to obtain the area of projections per section: 

macro “Measure Stack” {

      saveSettings;

      setOption("Stack position", true);

      for (n = 1; n <= nSlices; n++) {

              setSlice(n);

              run("Measure");

             }

             restoreSettings;

            }

Once the average area per section in μm^2^ was obtained, we multiplied the average by the number of sections (at 0.5 μm) per brain to obtain the dendritic volume in μm^3^.

To quantify microglia occupancy per unit area, we first converted the red (568) image into a black-and-white 8-bit image under ImageJ. The image was thresholded to highlight Iba1^+^ cell bodies and ramifications in red, and we divided the amount of red immunofluorescence by the unit area per section. To obtain microglial cell size (μm^2^), we took the same thresholded images and used the “Analyze particles” plugin in ImageJ with cell size defined from 0 to infinity and circularity from 0.00 to 1.00. The individual performing all immunofluorescent quantification was blinded to treatment groups and sex. 

### 2.5. P28 ProcartaPlex Mouse Hippocampal Cytokine and Chemokine Assay

P28 sham and IUGR mice (n = 4/group/sex) were sedated with ketamine and xylazine, and both hippocampi were dissected from bivalved whole brains and snap-frozen as one sample. We weighed hippocampi in 2 mL microcentrifuge tubes and added 500 μL of cell lysis buffer (EPX-99999-000, Thermo Fisher Scientific) per 100 mg tissue per sample. We homogenized all samples at 25 Hz for 0.5 min and centrifuged at 16,000× *g* for 10 min at 4 °C. We transferred supernatants to new microcentrifuge tubes and measured protein concentrations using BCA assay with protein standard concentrations ranging from 0.125 to 2 μg/μL (#23225, Thermo Fisher Scientific). Mouse cytokine and chemokine levels were measured using the 36-plex ProcartaPlex convenience panel 1A (Lot # 378502-001, EPXR360-26092-901, Thermo Fisher Scientific) following the manufacturer’s protocol in the MAGPIX L200 analyzer using the fluorescent-based detection system based on Luminex xMAP technology. In brief, serial dilutions of standard curves of all 36 analytes were prepared. Capture bead mixes were added to a 96-well plate and washed. Samples of 25 μL or standards plus 25 μL 1X universal assay buffer were added to each well and incubated at 600 rpm for 2 h at room temperature. Once washed, 25 μL of biotinylated detection antibody mix was added to each well and incubated at 600 rpm for 30 min at room temperature. Lastly, 50 μL streptavidin-PE was added and incubated at 600 rpm for 30 min at room temperature. A total of 120 μL reading buffer was added to the wells after shaking for 5 min, and the plate was read on the analyzer with settings at an acquisition volume of 50 μL, standard PMT reporter gain, and a minimum of 50 bead count.

Out of the 36 analytes, 22 were quantifiable based on the concentrations obtained. The concentrations of the samples were calculated by plotting the expected concentrations of the standards against the NET Mean Fluorescence Intensity (MFI) generated by each standard. We exported our run data in .csv format into the ProcartaPlex Analysis App on Thermo Fisher Connect: https://apps.thermofisher.com/apps/procartaplex (accessed on 14 April 2024) to generate the data. Both four- and five-parameter logistic (4PL/5PL) functions were available to fit the dose–response standard curves.

### 2.6. Statistics

All data were tested for normal distribution and equal variances prior to determining whether parametric or non-parametric tests could be used to determine statistical significance with StatView (SAS Institute Inc., CA, USA). If parametric, data were expressed as means ± SEM, and ANOVA with Fisher’s PLSD post hoc test was used for the four groups. If non-parametric, data were expressed as box-and-whisker plots with dark lines within the boxes denoting the median, lines of the outer boxes denoting lower and upper quartiles (the entire box is the interquartile range), and whiskers denoting the minimum and maximum values and Kruskal Wallis test was used for the four groups. A *p*-value ≤ 0.05 was declared as statistically significant. We used G*Power v3.1.9.7 to calculate the sample size of eight per group using a priori power analysis with ANOVA of four groups, noting an effect size of 0.6, α 0.05, and power 0.8.

## 3. Results

### 3.1. IUGR Preserved Principal Dendrite Lengths but Decreased Dendritic Branching and Volume of Hippocampal DG Neurons

**Figure 2:**
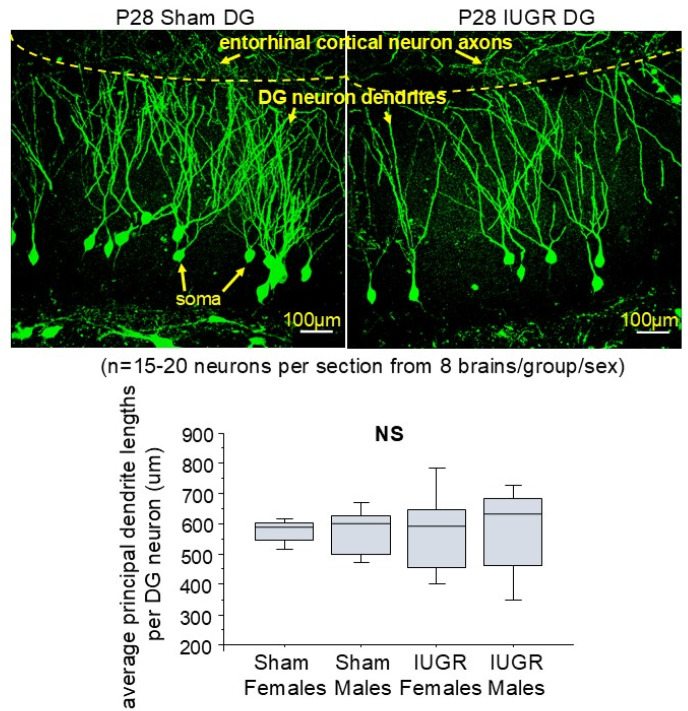
The average principal dendrite lengths per DG neuron (μm). Representative immunofluorescent photomicrograph images of the P28 sham and IUGR hippocampal DG are shown (n = 15–20 neurons per section from eight brains in both treatment groups separated by sex). The yellow dotted line denotes the anatomical border between entorhinal cortical neuron axons interfacing with DG neuron dendrites. DG neuron soma are seen at the base of dendrite projections. The box and whisker plots below the photomicrographs show that IUGR did not alter the average principal dendrite lengths per group and sex. NS = not statistically significant with the Kruskal Wallis test.

**Figure 3:**
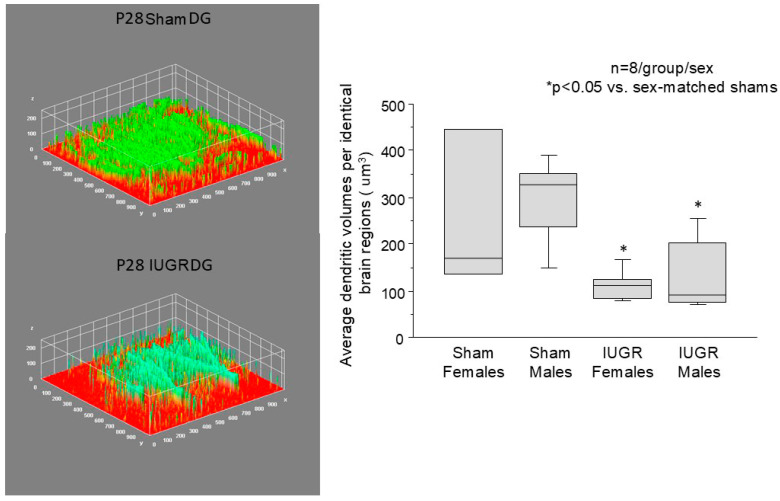
The average dendritic volumes per identical brain region (um^3^). Representative three-dimensional dendritic volume plots of P28 sham and IUGR hippocampal DG are shown (n = 15–20 neurons per section from eight brains in both treatment groups separated by sex). The box and whisker plots show that IUGR decreased the average dendritic volumes in both females and males compared to sex-matched shams. *p* < 0.05 in IUGR vs. sex-matched shams with the Kruskal Wallis test.

Figure 2 shows the dendrite morphology of P28 sham and IUGR hippocampal DG neurons. Axons emanating from the entorhinal cortical neurons send outgoing information to the hippocampal DG by synapsing with the dendrites of DG neurons. IUGR preserved the principal dendrite lengths, defined as the longest lengths between the soma and the entorhinal cortex/DG border, but decreased dendritic branching per DG neuron compared to the sham. This is validated in Figure 3 by the overall decreased dendritic volumes per identical brain regions. We saw that the number of DG neurons was also diminished in IUGR, potentially exacerbating the overall decrease in dendritic volumes. 

### 3.2. IUGR Augmented Microglial Number and Cell Size/Ramifications in the Hippocampal DG

**Figure 4:**
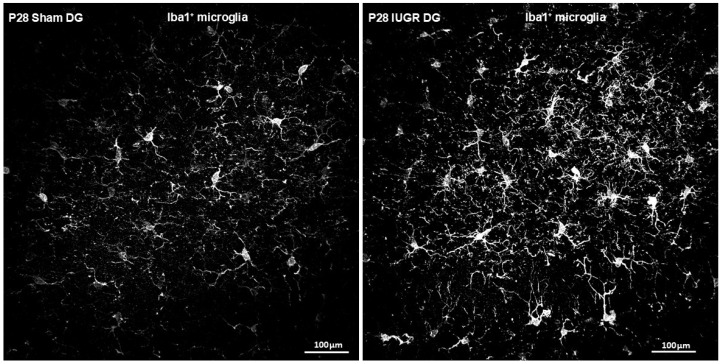
Representative photomicrographs of Iba1^+^ microglia staining in P28 sham and IUGR hippocampal DG (n = 8 brains per group and sex). Red staining has been rendered black and white for ease of viewing of microglial morphology.

**Figure 5:**
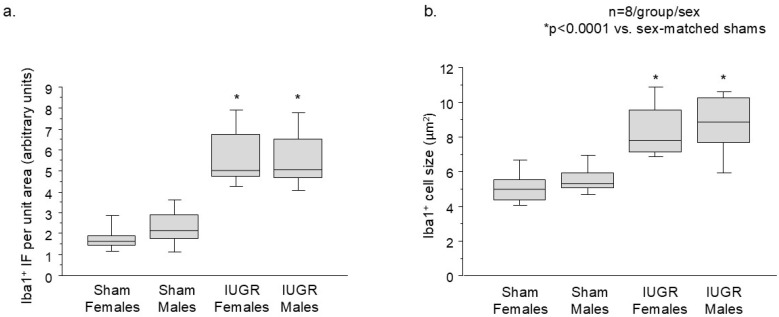
Quantification of Iba1^+^ immunofluorescence (IF) per unit area (arbitrary units, n = 8 brains per group and sex). (**a**) The box and whisker plots show that IUGR females and males had increased Iba1^+^ microglial IF per unit of hippocampal DG area, and (**b**) the Iba1^+^ cell sizes were larger compared to sex-matched shams. *p* < 0.0001 vs. sex-matched shams with Kruskal Wallis test.

**Figure 6:**
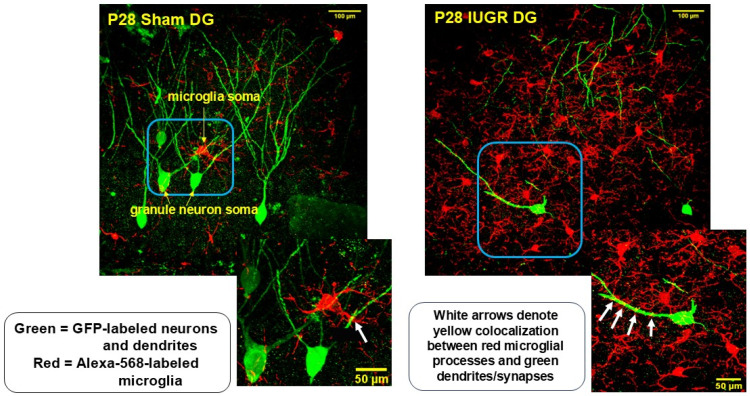
Representative immunofluorescent images of P28 sham and IUGR hippocampal DG green GFP-labeled DG neurons and their dendrites co-staining with red Iba1^+^ microglia. The higher magnification images (60x) below each lower magnification image (40x) show the yellow co-staining to highlight that red microglial processes are in direct contact with green DG dendrites/synapses.

Given microglia’s role in dendritic/synaptic pruning, we investigated their presence in the hippocampal DG. Using Iba1 as a mature microglial marker, we found that IUGR DG had increased microglial number as well as cell size and ramifications compared to sham DG (Figure 4 and Figure 5). Higher magnification revealed that red microglial ramifications were in direct contact with green dendrites, shown by the yellow co-localized synapses (Figure 6). 

### 3.3. IUGR Altered P28 Hippocampal Cytokine/Chemokine Profile in a Sex-Specific Manner

**Figure 7:**
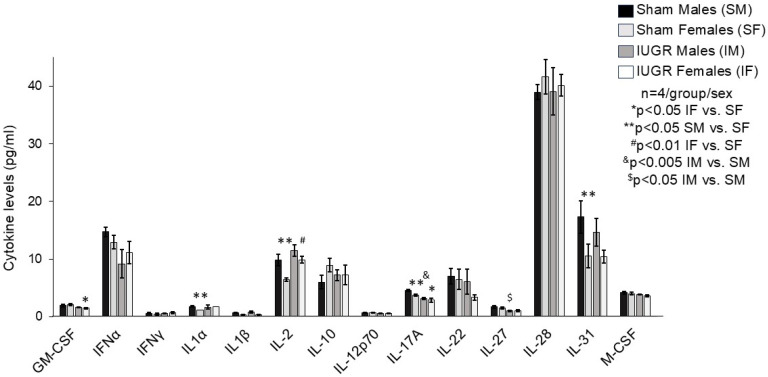
Quantification of P28 hippocampal chemokine levels in sham and IUGR (pg/mL, n = 4 hippocampi/group/sex). Black and dark gray bar graphs denote sham and IUGR males (SM, IM), respectively. Light gray and white bar graphs denote sham and IUGR females (SF, IF), respectively. Sham males and females exhibited sex-specific differences at baseline (** *p* < 0.05 SM vs. SF). All IUGR offspring decreased hippocampal IL-17A expression (^&^ *p* < 0.005 IM vs. SM and * *p* < 0.05 IF vs. SF). IUGR males additionally decreased IL-27 (^$^ *p* < 0.05 IM vs. SM), whereas IUGR females decreased GM-CSF (* *p* < 0.05 IF vs. SF) but increased IL-2 (^#^ *p* < 0.01 IF vs. SF). Statistical significance was determined by ANOVA with PLSD post hoc test.

**Figure 8:**
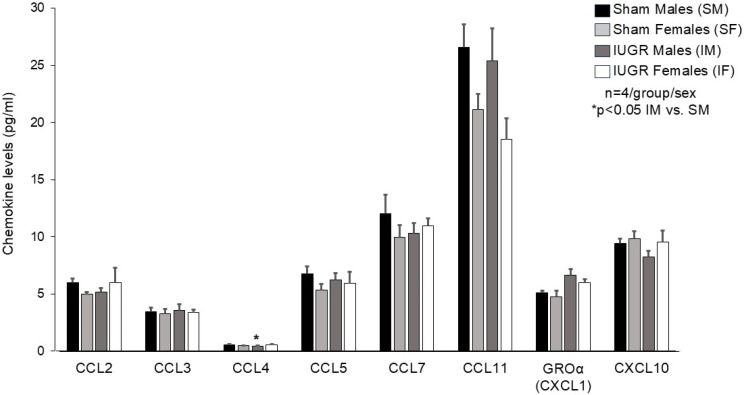
Quantification of P28 hippocampal chemokine levels in sham and IUGR (pg/mL, n = 4 hippocampi/group/sex). Black and dark gray bar graphs denote sham and IUGR males (SM, IM), respectively. Light gray and white bar graphs denote sham and IUGR females (SF, IF), respectively. IUGR males increased CCL4 compared to sham males (* *p* < 0.05). Statistical significance was determined by ANOVA with PLSD post hoc test.

The change in microglial morphology led us to investigate the hippocampal cytokine and chemokine milieu between the P28 sham and IUGR hippocampi. At baseline, sham males and females exhibited a sexually dimorphic cytokine profile such that sham females had decreased hippocampal IL-1α, IL-2, IL-17A, and IL-31 levels compared to sham males (Figure 7). No significant baseline differences are evident in hippocampal chemokine levels between sham males and females (Figure 8). One month after IUGR, IUGR males showed decreases in hippocampal IL-17A and IL-27 compared to sham males (Figure 7). IUGR males also decreased CCL4 (=MIP-1β) chemokine (Figure 8). IUGR females showed decreased hippocampal granulocyte-macrophage colony-stimulating factor (GM-CSF), a further decrease in IL-17A similar to IUGR males, but an increased IL-2 level (Figure 7). 

## 4. Discussion

IUGR-induced hippocampal DG neuronal alterations

Offspring born with IUGR are at an increased risk of neurocognitive disorders, particularly those affecting learning and memory [8,9]. The hippocampus is a medial cortical structure required for forming new memories and learned behaviors in vertebrates [20]. It is particularly vulnerable to chronic nutrient deficiency, such as that which occurs in IUGR, because of its protracted developmental timeline that begins in utero and continues through early childhood [21]. Animal models have previously shown that IUGR offspring have hippocampal neuron loss and abnormal neuronal morphology in both the DG and CA subregions in postnatal life [22,23]. These culminate in hippocampal volume loss, with a greater volume loss directly correlating with worse memory deficits. Human IUGR infants also exhibit greater memory loss when the hippocampal volumes are the smallest when quantified with magnetic resonance imaging [24,25]. 

Our laboratory has a special interest in understanding the fetal origin of postnatal hippocampal neuronal alterations in a translational model of HDP/UPI/IUGR in mice. We have previously published evidence that HDP/UPI induced at embryonic day (E) 12.5 (in a 20-day pregnancy of C57BL/6J mice) causes premature embryonic hippocampal DG neurogenesis at E15.5, which persists to delivery [14]. Concurrently, we saw neural stem cell (NSC) depletion that continued through delivery [14], suggesting that NSCs exit the cell cycle early to commit to a neuronal lineage in HDP/UPI. Our current study strives to delineate the postnatal outcomes of these prematurely differentiated DG neuronal progenitors by examining their postnatal morphology, including their dendrite formation. We have focused on DG neurons because they are the first neurons to receive incoming information from the entorhinal cortex into the hippocampus proper for memory formation. Any perturbation in neuron maturation could set the stage for future learning and memory impairment.

Our data show that one month after HDP/UPI, IUGR hippocampal DG neurons preserved their principal dendrite lengths but had decreased dendritic branching and dendritic volumes in both sexes compared to shams. In a chronic placental insufficiency model induced by unilateral uterine artery ligation in guinea pigs at mid-gestation, Dieni and Rees also found that DG neurons from fetuses delivered close to term gestation had reduced dendritic outgrowth [11]. In a rat prenatal stress (PNS) model, prepubertal pups born to mothers with PNS demonstrated decreased dendritic length and complexity in males and females. Collectively, these data suggest that alterations in dendritic morphology are common consequences of adverse intrauterine environments. Our model, however, adds new insight by showing that, even when premature embryonic DG neurogenesis occurs in HDP/UPI, possibly as a compensatory survival mechanism, abnormal postnatal dendritogenesis still occurs in a cell-autonomous fashion even after the UPI insult is removed. The longer-term consequence of such decreased dendritogenesis in our model is learning and memory impairment, as previously tested by novel object recognition and fear conditioning tests [14]. IUGR females fare worse than IUGR males in these memory tasks at 2–6 months of age, and IUGR females have the smallest postnatal hippocampal volumes at P18 and P40 prior to overt memory deficits [14]. We are certainly intrigued by the sexually dimorphic neurologic mouse phenotype because we know that human IUGR infants also exhibit differing neurological susceptibilities in various domains of neurocognitive and neuropsychiatric functions [26,27,28]. Apart from genetic sex and hormonal influences, we posit that IUGR females may fare worse in our model than IUGR males due to their slower catch-up growth in the first 2.5 months of life. IUGR males catch up to sham males’ weights by P28, whereas IUGR females catch up at P77, a time in which significant dendritogenesis and synaptogenesis occur in these DG neurons. We know that poor catch-up growth, especially in brain growth, is a well-recognized risk factor for future neurological compromise after IUGR [29,30].

IUGR-induced hippocampal DG microglial alterations

Seeing decreased DG neuron dendritic complexity, we sought to understand whether such altered morphology might involve microglia, which are tissue-resident macrophages responsible for multiple functions during normal brain development. Importantly, the role microglia play in the formation and function of neuronal circuits has gained significant attention in recent years [31,32]. In the rodent hippocampus, microglia precursors arrive at the primitive hippocampal formation from the extra-embryonic yolk sac beginning at from E9.5 to E14, further arranging themselves along subregions and layers according to an ontogenic program that mimics the neuronal differentiation patterns [33]. Microglia follow an outside-to-inside distribution, starting from the hippocampal fissure, first reaching the main layers of the cornu Ammonis’ horn and then to the DG. They then differentiate from birth to ~P18, progressing from a round or pleomorphic shape with few filopodia to an oval or slightly elongated shape that is characteristic of primitive ramified microglia. At P12, mature homeostatic microglia are noted to be present with fully developed ramifications [33]. 

Microglia have been found to mature alongside neuronal circuits in a bidirectionally regulated process both in embryonic and postnatal times such that they participate in neurogenesis, synaptogenesis, and circuit refinement through synaptic pruning and myelination [19]. At the time of examination for our current study, synaptic patterning should be a predominant task of microglia such that stronger neuronal connections are reinforced while weaker synapses are eliminated. Our data show that IUGR hippocampal DG of both sexes has an increased Iba1^+^ microglia presence along with increased cell sizes and ramifications compared to those of sham hippocampal DG. Both sham and IUGR microglial processes make direct contact with neuronal dendrites and likely the synapses for synaptic modulation. At this juncture, we do not yet know whether these direct microglial-dendritic interactions are reinforcing or eliminating synaptic connections. Both increased and decreased developmental synaptic pruning in the hippocampus have been associated with future neurodevelopmental disorders [31,34]. We are in active pursuit of understanding this interaction by determining the homeostatic versus activated state of the microglia as well as enumerating the synaptic boutons that exist between sham and IUGR DG dendrites.

IUGR-induced hippocampal DG neuronal–microglial interactions

Lastly, because the microenvironment within the hippocampus drives the activity of neuron–microglial interactions, we pursued an analysis of a selected number of pro-inflammatory and anti-inflammatory cytokines and chemokines that may exist within the P28 sham and IUGR hippocampi. Not unexpectedly, we noted a sexually divergent hippocampal cytokine profile between sham males and females at baseline. In general, sham females at P28 exhibited a less pro-inflammatory milieu with decreased IL-1α, IL-2, IL-17A, and IL 31 expression compared to P28 sham males. This is because microglia are known to display a dynamic sexual dimorphism in their number and morphology throughout development [35]. As mentioned before, the rodent brain initially develops round or amoeboid-shaped microglia. As the brain matures over time, microglia develop a thicker or longer and ramified morphology in several brain regions regardless of sex. Interestingly, the rate of microglial colonization and morphological development differs between males and females such that males have more mature microglia early in postnatal development and possibly a more proinflammatory profile, whereas females possess more microglia with activated morphology starting around early puberty and in adulthood [36]. 

After IUGR, males and females displayed varying responses to the in utero insult. All IUGR offspring displayed decreased hippocampal IL-17A expression. IUGR males additionally displayed decreased IL-27 and CCL-4 compared to IUGR females, which displayed decreased GM-CSF but increased IL-2. IL-17A is a pro-inflammatory cytokine that can disrupt hippocampal long-term potentiation and inhibit neurogenesis in the adult DG with over-expression, particularly in inflammatory conditions [37]. The role of IL-17A under a non-inflammatory physiological condition is less defined. However, Liu et al. showed that genetic deletion of IL-17 increased the number of adult-born neurons in the DG [38]. IL-17 deletion also altered the cytokine network, facilitated basal excitatory synaptic transmission, enhanced intrinsic neuronal excitability, and increased the expression of proneuronal genes in neuronal progenitor cells [38]. Suffice it to say that this phenotype is particularly intriguing to us because we have previously found that HDP/UPI induces embryonic NSC depletion. The fate of these NSCs in postnatal life represents another active area of investigation in our laboratory. An overall decrease in IL-17 expression in IUGR offspring could signify a possible mechanism for postnatal NSCs to regain their proliferative ability in order to generate more neuronal progenitors as the brain continues to develop postnatally. In addition to the role of IL-17 on neuronal progenitor cells, IL-17A has also been shown to stimulate glial cells to secrete brain-derived neurotrophic factor (BDNF) to support synaptic transmission [39]. An overall decrease in IL-17 with an accompanying decrease in BDNF after IUGR could seriously affect eventual hippocampal neuronal function, as BDNF is a key neurotrophin indispensable for learning and memory [40,41,42].

IL-27 is a pleiotropic cytokine that has both pro- and anti-inflammatory effects [43]. Evidence is accumulating that IL-27 has neuroprotective activities in the brain and retina. IL-27 is secreted from and binds to microglia, astrocytes, and neurons to promote neuronal survival by regulating pro- and anti-inflammatory cytokines, neuroinflammatory pathways, oxidative stress, and apoptosis [44,45]. However, IL-27 can have the opposite effect and induce inflammation and cell death in certain situations [46]. CCL-4 is the only chemokine that was affected by IUGR, and this occurred in males only. The significance of a decrease in hippocampal CCL-4 expression in IUGR is less clear because CCL-4 is such a ubiquitous chemokine after tissue injury in many organs [47,48,49,50]. A study performed by Pala et al. has found that elevated levels of proinflammatory factors, such as CX3CL1 and CCL-4 (or MIP-1β), along with other inflammatory cytokines such as TNF-α, IL-1β, and IFN-γ, and increased HIF-1α levels, are associated with IUGR attributed to a hypoxic amniotic environment [51]. Whether the decrease in IUGR offspring hippocampal CCL-4 expression represents a feedback downregulation is unknown at this time.

The innate immune system stimulating cytokine GM-CSF is another cytokine that has both pro- and anti-inflammatory activities. The use of exogenous GM-CSF as a general cognition enhancer despite its pro-inflammatory/immune modulatory activity has gained significant attention, particularly in brain disorders that are associated with chronic inflammation, such as Down syndrome (DS), Alzheimer’s disease (AD), and COVID-19 [52,53]. Multiple authors have discovered that GM-CSF treatment reduced cognitive deficits in mouse models of AD and even during normal aging [54,55]. The beneficial effects of GM-CSF on recovery from neuronal damage are postulated to occur via modulation of apoptosis-related genes, promotion of axonal growth, and through it acting as a growth factor for NSCs, to name a few mechanisms [56,57,58]. Treatment with GM-CSF has also improved learning, memory, and neuropathology in the Dp16 mouse model of DS and in wild-type mice [53]. As such, the decrease seen in IUGR females likely affects multiple pathways of neuronal function, placing them at increased risk of learning and memory deficits. Lastly, IUGR females increased their hippocampal IL-2 expression. IL-2, the cytokine also known as T-cell growth factor, has multiple immunoregulatory functions and biological properties not only related to T cells. In the past decade, substantial accumulated evidence suggests that IL-2 is also a neuroregulatory cytokine that can improve synaptic plasticity and rescue spine density in the hippocampus [59]; therefore, upregulating IL-2 expression could be beneficial in IUGR. 

## 5. Conclusions

The most important message of our study is that the learning and memory deficits seen after IUGR likely represent a complex interplay amongst multiple cell types and their neuroregulatory factors. Our study has begun to dissect such interplay between hippocampal DG neurons and microglia after IUGR, which has certainly opened up more questions than current answers. Our study is mainly limited by the descriptive nature of our findings. Our hope is to validate some of these findings by determining whether microglia are actively engulfing synapses, whether microglial precursor migration from the yolk sac is initially perturbed by HDP/UPI, and whether microglial proliferation or differentiation drives the current morphological changes. Until we understand how these basic developmental processes are affected by IUGR, we will not be able to move forward to further dissect the mechanisms of disease.

## Data Availability

The raw data supporting the conclusions of this article will be made available by the authors upon request.
